# Providing recurrence risk counselling for parents after diagnosis of a serious genetic condition caused by an apparently de novo mutation in their child: a qualitative investigation of the PREGCARE strategy with UK clinical genetics practitioners

**DOI:** 10.1136/jmg-2023-109183

**Published:** 2023-03-17

**Authors:** Alison C Kay, Jonathan Wells, Nina Hallowell, Anne Goriely

**Affiliations:** 1 MRC-Weatherall Institute of Molecular Medicine, Radcliffe Department of Medicine, University of Oxford, Oxford, UK; 2 NIHR Oxford Biomedical Research Centre, Oxford, UK; 3 The Ethox Centre and Wellcome Centre for Ethics and Humanities, Nuffield Department of Population Health, University of Oxford, Oxford, UK

**Keywords:** genetic counseling, reproductive medicine, genomics

## Abstract

**Background:**

Diagnosis of a child with a genetic condition leads to parents asking whether there is a risk the condition could occur again with future pregnancies. If the cause is identified as an apparent de novo mutation (DNM), couples are currently given a generic, population average, recurrence risk of ~1%–2%, depending on the condition. Although DNMs usually arise as one-off events, they can also originate through the process of mosaicism in either parent; in this instance, the DNM is present in multiple germ cells and the actual recurrence risk could theoretically be as high as 50%.

**Methods:**

Our qualitative interview study examined the views and reflections on current practice provided by UK practitioners working in clinical genetics (n=20) regarding the potential impact of PREcision Genetic Counselling And REproduction (PREGCARE)—a new preconception personalised recurrence risk assessment strategy.

**Results:**

Those interviewed regarded PREGCARE as a very useful addition to risk management, especially for cases where it revised the risk downwards or clarified that a couple’s personalised recurrence risk meets National Health Service thresholds for non-invasive prenatal testing, otherwise inaccessible based on the generic DNM recurrence risk.

**Conclusion:**

Participants said it could release some couples requiring reassurance from undergoing unnecessary invasive testing in future pregnancies. However, they regarded mosaicism and PREGCARE as complex concepts to communicate, requiring further training and additional appointment time for pre-test genetic counselling to prepare couples for all the possible outcomes of a personalised risk assessment, including potentially identifying the parental origin of the DNM, and to ensure informed consent.

**WHAT IS ALREADY KNOWN ON THIS TOPIC**

- De novo mutations (DNMs) cause developmental disorders in ~1 in 295 live births.
- While case reports of recurrence may have been published for a specific condition, they do not provide an estimate of the mosaic risk to other couples, unless they are systematic unbiased studies of large cohorts.
- The generic recurrence risk quoted to couples with an affected child of ~1%–2% represents a population average and is not accurate for any given couple.
- In ~10% of such families, one of the parents is actually mosaic for the DNM which carries an increased recurrence risk of up to 50%.
- PREcision Genetic Counselling And REproduction (PREGCARE) is a new genomic strategy for refining and personalising the recurrence risk of genetic conditions associated with a DNM.

**WHAT THIS STUDY ADDS**

- Insight into clinical genetics practitioners’ views and experiences of offering couples counselling on recurrence risk following the birth of a child with a disorder caused by a DNM and the potential benefits of the PREGCARE strategy to their practice.

**HOW THIS STUDY MIGHT AFFECT RESEARCH, PRACTICE OR POLICY**

- The study suggests that PREGCARE provides personalised evidence which can add clarity to DNM recurrence risk management and can support couples’ reproductive decision-making *prior* to a new pregnancy.
- Implementation of the strategy in a clinical setting will require additional training for practitioners regarding communication of the testing strategy and the interpretation of personalised recurrence risks for couples considering further pregnancies.

## Introduction

The birth of a child with a serious clinical disorder (eg, with complex learning disabilities, severe physical impairment, shortened life span) to a healthy couple with no previous family history is a life-changing event. De novo mutations (DNMs) cause developmental disorders in ~1 in 295 live births^1^ and when a DNM is identified as the cause, in the majority of cases this is a one-off event, which occured either in a single gamete from one of the parents (egg or sperm), or early in the child’s own embryonic life. In such cases, the recurrence in a future pregnancy is essentially negligible. However, if the DNM first arises at an early timepoint in the embryonic development of one of the parents, it can be present in multiple gonadal cells (‘gonadal mosaicism’). In this situation, the recurrence risk can be theoretically as high as 50%.^2^ Not knowing the specific circumstances of individual couples, practitioners currently rely on a population-average risk estimate of the DNM occurring in a future pregnancy of ~1%–2%. This *generic recurrence risk* does not separate those couples with negligible risk from those with a higher risk. As a result, couples are asked to make potentially life changing reproductive decisions with information that is almost always incorrect and potentially misleading.

**Figure 1:**
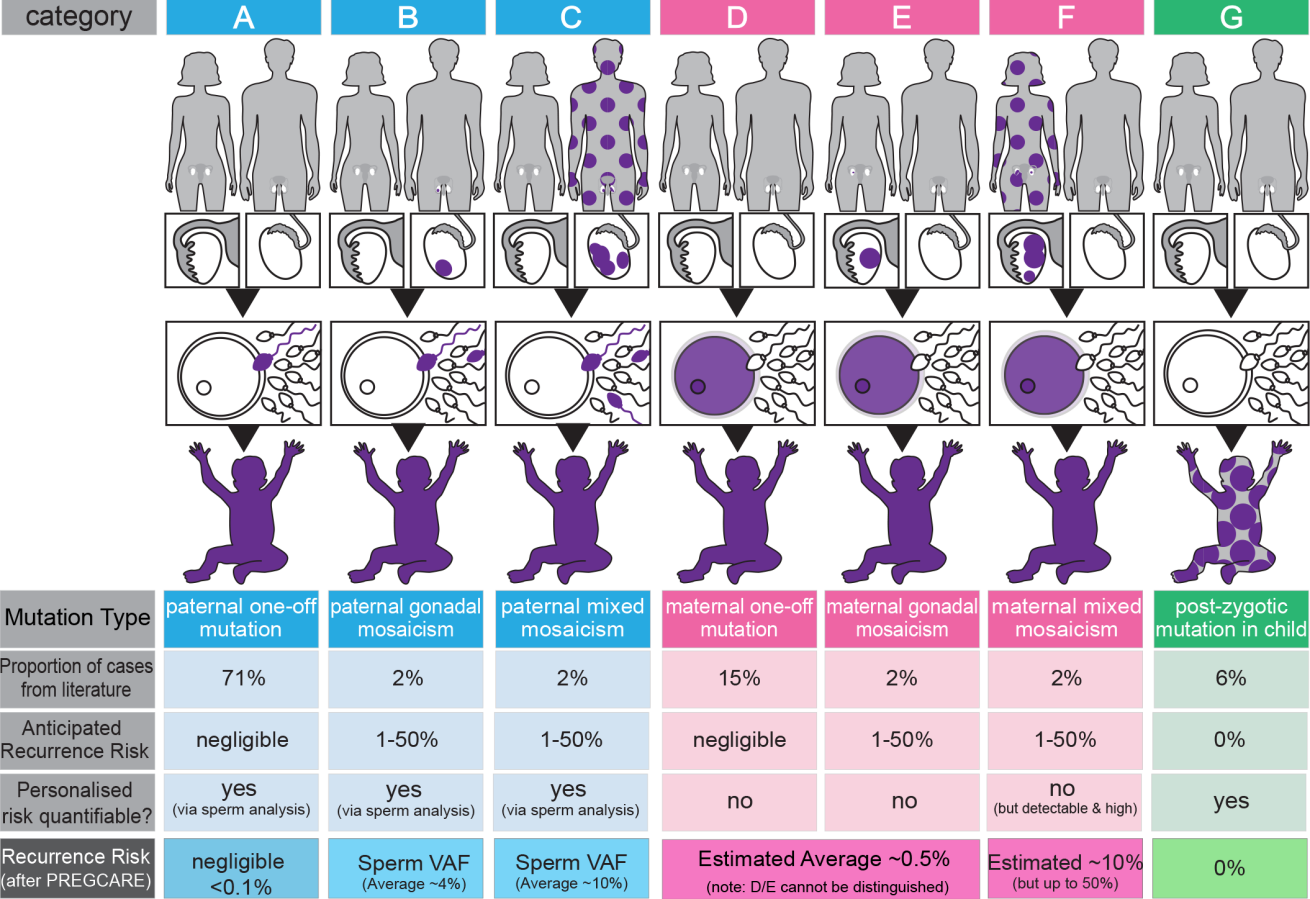
Overview of the PREcision Genetic Counselling And REproduction (PREGCARE) strategy and stratification of de novo mutation (DNM) recurrence risk into seven different categories. By establishing the origin (paternal (blue), maternal (pink) or postzygotic (proband, green)) and the timing of the mutational events (purple colour indicates mutant cells), it is possible to stratify individual families into different categories that are associated with widely different recurrence risks (see ‘anticipated recurrence risk’ in the figure). The proportion of cases in each category can be estimated using data from the literature. Four of the seven categories (ie, categories B, C, F and G) involve mosaic presentations and can be identified by deep sequencing of the collected tissues from the family trio. Furthermore, analysis of a sperm sample for paternal cases allows direct quantification of the risk to another pregnancy (ie, variant allele frequency (VAF) of the DNM in the semen sample). By singling out these mosaic families, the remaining (mosaic-negative by deep sequencing) categories (A, D, E) have a reduced risk of recurrence estimated to be ~0.1% (>10-fold reduction over the generic 1%–2% risk) (see Bernkopf *et al* 2 for details). The last row of the figure represents an overview of the refinement of risk generated during the PREGCARE strategy. Analysis of the DNM parental origin via long-read sequencing allows to further refine the risk for the mosaic-negative categories and reassure the majority of families, as category A (one-off paternal, 71% of cases) is associated with a negligible risk, estimated to be below 0.1% depending on the exact limit of detection for the specific custom assay; note that categories D and E cannot be distinguished from one another because it is not possible to access maternal oocytes and the risk for a DNM of proven maternal origin is estimated to be reduced modestly (~2×–8×) compared with the population with generic risk. Likewise, the risk associated with category F (mixed maternal mosaicism) cannot be quantified but is likely to be ‘high’ and can be estimated to be on average ~10% (for further details and references, see Bernkopf *et al* 2). Figure adapted from Bernkopf *et al*.^2^

The UK-led PREcision Genetic Counselling And REproduction (PREGCARE) study developed a systematic strategy for providing families with a *personalised risk assessment* following the birth of a child with a genetic condition caused by DNM.^2^ This relies on analysis of tissues (blood, saliva, buccal swabs, urine and sperm) from child-parent trios and stratifying each of the 60 families recruited into one of 7 categories associated with different recurrence risks (figure 1). The experimental strategy involves ultra-deep sequencing of the tissues to identify cases of occult mosaicism, followed by haplotyping of the DNM for the mosaic-negative families to determine parent-of-origin. Individual clinical reports were returned to the referring clinicians who informed the families of their results. The PREGCARE study was able to provide a refined risk that, in all cases, differed from the 1% to 2% generic risk originally given to couples. In ~10% of recruited families, the ultra-deep sequencing identified evidence of mosaicism in one of the parents that had been missed on previous routine analysis—associated with an increased risk of recurrence—but for the other 90% of couples the risk was reduced.^2^

The PREGCARE strategy has the potential to transform genetic counselling practice by allowing couples to make future reproductive decisions based on personalised information. However, before the argument can be made that this should be implemented as part of routine clinical care, it is important to consider clinical genetics professional’s experiences of providing recurrence risk information to couples and their views on the current challenges and unmet needs as well as the potential impact of introducing a personalised approach for service provision and patient outcomes.^3^

Existing literature shows a difference in lay and health professionals’ perspectives on how reproductive risks are perceived and received.^4 5^ Pregnant women’s perception of population-based risk is driven more by their interpretation of their subjective risk—influenced by experience, personality and beliefs—and feelings about the acceptability of the risk being considered, than the objective probability of an affected pregnancy.^4–7^ However, if the availability of personalised, evidence-based, risk information appears an attractive option to counselling practice, it may complicate this picture and create new challenges.

## Methods

Research participants (n=20) were recruited via advertisement to professional associations: (British Society of Genetic Medicine and the Association of Genetic Counsellors and Nurses). All had experience of counselling couples about prenatal options following diagnosis of conditions caused by a DNM in their child. Fourteen clinical geneticists (CG) and 6 registered genetic counsellors (GCr) were recruited, from 15 NHS Trusts across the UK. Twelve out of 20 participants had direct experience of referring couples into the original PREGCARE study. Fourteen out of 20 participants were women, including 1 male GCr, reflecting the gender composition of the profession.^3^

Semi-structured interviews (duration ~60 min) were conducted remotely via Microsoft Teams between February and June 2022, beginning approximately 10 months after the PREGCARE research study enrolled the last family in March 2021. Interviews covered included: interviewee’s current practice in providing recurrence risk information; views on the usefulness of generic recurrence risk; views of, or experience with, PREGCARE and reflections on the practicalities of introducing PREGCARE into clinical practice. A qualitative data analysis software package, NVivo, was used to manage the data generated and Reflexive Thematic Analysis was used to analyse the data.^8 9^ The analysis revealed a number of themes reflecting practitioners’ experiences including: *responsibility; the reassurance gap and the communication challenge for mosaicism* and *tools of reassurance*. The latter two are reported below. The Standards for Reporting Qualitative Research reporting guidelines were used.^10^

## Results

The analysis suggested there were few discernible differences between the responses of CG participant number and GCr participant number.

### The reassurance gap and issues for risk communication

Clinical genetics professionals described a tension between the generic recurrence risk currently provided to couples and couples’ subjective perceptions of the risk of a recurrence in a future pregnancy. Thus, providing reassurance was described as challenging, especially for those couples wanting total reassurance. Practitioners thought it a very difficult situation for couples who do not feel reassured after receiving a generic recurrence risk, although small (1%–2%), acknowledging that they are left with underlying uncertainty. Practitioners were aware that the generic risk figure is not accurate for any given couple (and may vary depending on the specific condition) and that the potential consequences of recurrence—having an affected pregnancy—was not an abstract fear because these couples had already had a child with a life-changing genetic condition. As GCr19 said: *“It’s still kind of leaving them I guess a bit in limbo because we're saying, ‘It’s most likely low risk but there is a chance’…”* (table 1, GCr07)

**Table 1:** The reassurance gap

*GCr07*	*“I think there’s an inevitable tension between the experiential and the technical, that you generally want to reassure someone that something is low risk and that this is good news and want to take away perhaps some of that fear or anxiety that they might be living with. Sometimes those two things just don’t meet…”*
*CG02*	*“I had been reassuring to some couples…and I might have just been lucky…”*
*GCr20*	*“…it* [a subsequent affected pregnancy] *took us all by massive surprise and was quite traumatic as well. Even though you review your correspondence, you wrote all the right things, everything. All the advice they were given was correct. It still makes your heart stop when you realise that a baby has had a recurrence and the implications of that on the patient, who had to have a late termination of pregnancy, and who obviously was absolutely devastated.”*
*CG10*	*“I guess you always worry that you haven’t done your research well enough or with these very rare groups of disorders that I’ve missed something…You know, do I need to scour the literature every time for a new rare de novo disorder to make sure that actually for some reason there’s not a higher published recurrence risk with that disorder? I guess it’s a little bit of uncertainty but normally, as it flashes in my mind, I just provide a generalised recurrence risk.”*
*GCr17*	*“I think the real difficulty with the situation at the moment is there will be a lot of couples where actually there is no risk, but you can’t say that, and then so everyone falls into the 1% whereas by doing this* [PREGCARE] *you’ve taken away the 0% bit, so people at least know that they are in the category of it could happen again.”*
*CG10*	*“These things, for interested couples, you might throw some of those facts out there but those are incredibly complex pieces of information that I’m just throwing out there that need a lot of unpacking. I only understand that after many years…”*
*GCr19*	*“I think if you’re explaining about gonadal mosaicism and explaining why the risk is not kind of zero, it’s really confusing. It’s really difficult to explain.”*
*CG15*	*“I guess I’m not doing…there’s not enough time to, and it doesn’t help their understanding, I don’t think, when they’re first hearing about mosaicism that day, about the technicalities of every different type of…mosaicism”*
*GC12*	*“Certainly, when we are doing testing in the first place to try and find a cause of their child’s condition, we often are talking about the fact that it might be a recessive condition and the recurrence risk is twenty-five per cent…”*

Practitioners said their limited experience of DNM recurrence bolstered their perception of the recurrence risk as ‘low’, often drawing comparisons with counselling about autosomal recessive inheritance in which unaffected ‘carrier’ parents have a 25% chance of having an affected child. They wondered whether they were lucky in not having a recurrence in their own patients yet, as CG16 reflected: *“Sometimes. I think experience empowers you to feel maybe overconfident. You know, I’ve yet to be, touch wood, I’ve yet to be caught out in my career”*. GCr20, whose patient had experienced a recurrence, recounted a very challenging experience for the team because, “*it took us all by massive surprise and was quite traumatic as well*”. It was an experience for which they felt unprepared (table 1, GCr02, GCr20).

Although some participants said, based on the comparably low DNM recurrence risk, that they were comfortable providing couples with a generic risk, others described generic risks as ‘*fudge figures*’ (CG08) and feared giving couples ‘*wildly inaccurate risks*’ (table 1, CG02). A general air of caution was expressed around the accuracy of the generic risk figure given, usually 1%–2%, with some reflecting that it was better not to focus on the numbers and that the practitioner should check the literature with each case for new information: *“I try to avoid numbers because I think a lot of the numbers are a guess in the first place”* (CG08) (table 1, GCr10).

In this context, PREGCARE was described as offering the possibility of more clarity, as GCr19 said, it ‘*certainty feels more comfortable*’ (table 1). However, when asked to consider a PREGCARE outcome identifying the maternal origin of a DNM (ie, maternal mosaicism, categories D, E, F on figure 1), for which precise quantification is not yet scientifically possible due to the inaccessibility of the oocytes (see legend to figure 1), the practitioners interviewed were ambivalent as to whether this was a gain. Participants thought identifying maternal mosaicism could be helpful in terms of risk management and accessing interventions, but expressed concern about counselling patients, describing this outcome as harder to counsel, not least because the wider margins of risk in this situation could be seen as *“…an increased amount of entropy, if anything, I would find that tough…tough to communicate”* (CG15) (table 1, GCr17).

Despite the potential benefits of a personalised approach, the idea of changing the DNM risk message currently given to couples was seen as challenging. Practitioners said they usually give mosaicism, especially somatic mosaicism, a brief and simplified explanation in clinic because it is complicated to understand (table 1, CG10, CG15, GCr19). The ‘out of the blue’ origin of DNMs and the associated ‘no one’s fault’ messaging they currently deliver was felt to provide relief to couples, who are usually aware of other potential genetic inheritance patterns (carrying higher standard recurrence risks): *“So, they may feel some level of relief that it’s not something that’s inherited. It’s not something where they might be carriers and there’s a one in four recurrence”* (GCr20) (table 1, GCr12). Subsequentially, deconstructing this to explain mosaicism and PREGCARE, which explicitly outlines the parental origin of the causative mutation in their child, was seen by some participants as challenging, requiring *“…more time and more appointments because you’re doing another round of testing with some lengthy explanations”* (GCr07) with *“too much background required to explain”* (CG15).

### Tools of reassurance and justification for prenatal procedures

Conveying thoroughness and being able to offer couples an action plan was seen as a valuable tool of reassurance. Interviewees said that in current National Health Service (NHS) practice actions are limited to informing couples regarding their ‘options’, which due to risk-specified access thresholds for accessing non-invasive options on the NHS (non-invasive prenatal testing (NIPT); NIPD; PGT-M), consist of invasive prenatal testing (PNT) in the next pregnancy. In many cases, PNT was not seen as medically necessary by interviewees, instead the offer and undertaking of PNT was seen as primarily an anxiety-reducing or anxiety-reassuring exercise, as CG14 said: *“…if it’s 1% or less, then I will arrange it for reassurance but personally I don’t think it’s necessary”* (table 2, GCr19). If a couple did not return to clinic to discuss prenatal options in the next pregnancy, practitioners took an optimistic stance: “*I take it that they’re therefore reassured”* (CG10), although they added that with no follow-up it was unknown “*if they just decided not to have any more”* (table 2, CG18)).

**Table 2:** Tools of reassurance

*GCr19*	*“…generally, the focus is on reassurance, which is yes, different to the other prenatal testing that we are doing.”*
*CG18*	*“…to have an invasive test where the miscarriage risk and the recurrence risk are the same, is such a difficult decision for people to make.”*
*GCr01*	*“I mean obviously it depends on their moral belief…their morals, their values, their religious beliefs, all different reasons to whether they would want invasive, and you know whether they would consider termination.”*
*GCr20*	*“Some couples will choose the NIPD route because it’s a non-invasive test and they don’t want to put a pregnancy at risk from a needle going in from an invasive test. It means that they… but they need to pay for it, so it’s that kind of balance*.”
*CG10*	*“They are reassured that we’ve applied the best of our knowledge and the best of genetic testing technologies to their situation and given them a figure.”*
*GCr07*	*“…we could open up other options of testing for them. For example, will they then meet the criteria for NIPD which, for some people, would be huge. You know that would be so beneficial if they could access that rather than having an invasive procedure, especially if it’s someone who is perhaps later in maternal age, pregnancies are becoming more precious, or again if there are religious issues or just personal feelings around termination.”*
*CG18*	*“It would make a big difference to the people that do have it* [PREGCARE] *and you would avoid miscarriages from invasive procedures for certain, which in itself is a huge thing and the cost of having a CVS is significant as well. There are so many good things about avoiding an invasive test.”*
*CG08*	*“Some of them would be that there’s pretty much 0% chance risk of it happening again and so then we’ve got to be bold and say to people, ‘Actually, you don’t need prenatal’. That’s the whole point of it, it’s not to put people through that difficult process. Yes, you’ve got to then be bold and say, ‘You don’t need it’.”*
*CG08*	*“If at the point of a child being diagnosed, if you then get accurate recurrence figures in the next few months afterwards, then it may be that they don’t need another appointment and another appointment and maybe they don’t get offered prenatal diagnosis.”*
*CG13*	*“The other thing is the acceptability, you know, if patients will find it acceptable to have so many samples taken. They will have to be really motivated for that otherwise they just might give up. They sometimes don’t turn up for an appointment when we just have to take a blood sample.”*
*GCr20*	*“It was so interesting because when I listed the samples that were needed, I thought that might put them off or I thought that they might worry about that, but they absolutely didn’t. They were quite happy to provide anything that would help to work things through.”*
*CG08*	*“If it’s high risk but not quantifiable, then you’re back into the fudge figure, so I suppose really, you’d just be saying to the people that, ‘We’re almost back to where we started, that there is a risk, but we don’t know exactly how high…’.”*
*CG14*	*“I’m doing it* [giving generic recurrence risk] *at the same time as the diagnosis, to be fair, usually they're focused on the diagnosis…So, actually, the recurrence risk in that situation is secondary…”*
*GCr19*	*“The logistics of the sort of urgent ad hoc contact with patients and midwives and FMUs and just the logistics of doing all of that and often doing it quite quickly if necessary…most often they come in around 10 or 11 weeks and you do have to get things, you know, obviously done quickly and that means that patients are potentially having less time to make those decisions as well.”*
*CG11*	*“So, you want to get it done* [PREGCARE] *before they’re planning their next pregnancy but the last thing you want to do is bring additional trauma to a couple who may well be in the midst of very deep grieving.”*
*CG10*	*“…a couple would need to understand all the ins and outs of those seven possible results and the negatives of the path that they are embarking down and the potential parent of origin issue. There is a lot of complex pre-test counselling there to be done.”*

Although, the importance of patient-centred practice and being empathetic to parents’ need for reassurance were justifications for further testing, not all participants were entirely comfortable with providing invasive testing: *“They’re going down an invasive testing route on a baby that looks normal on the scan…”* (CG11), nor directing couples to expensive non-invasive testing outside the NHS, as CG06 put it: *“…that’s the worry, that people go off and spend money they shouldn’t, they don’t need to spend* [on private NIPT]*”* (table 2, GCr06).

Interviewees were also aware that even presenting PNT as an option, places an evaluative burden on a couple, especially as access to tests, particularly invasive tests, often requires an expressed intention to terminate that pregnancy if a recurrence is identified (table 2, GCr01, CG18, GCr20). In this light, PREGCARE was perceived as a way of addressing the reassurance gap, conveying thoroughness and providing an action plan without the couple having to risk a current pregnancy or grapple with the acceptability of negative outcomes: *“I think it’s all about getting as much information as we can for people without putting a current or potential pregnancy at any risk”* (CG09) (table 1, GCr10).

PREGCARE was regarded as particularly ‘*beneficial*’ (GCr07) in cases where the personalised recurrence risk was identified as 10% or higher, the current threshold for eligibility to funded PGT-M in the NHS. Participants hoped that in such circumstances, in meeting the threshold for accessing NIPT on the NHS, this would become available to couples with a DNM risk. Where a personalised recurrence risk was lower than the generic recurrence risk, this was regarded as a ‘good news’ result and participants thought couples might feel less inclined to undergo PNT or to pay for a non-invasive test (table 2, GCr07, CG18). Furthermore, they wondered, if the PREGCARE assessment returned a personalised recurrence risk below the generic recurrence risk, this would be evidence to support the practitioner being more directive and not offering or dissuading the couple from PNT (table 2, CG08).

However, despite perceiving these benefits to risk management, some interviewees were uncertain as to whether couples would be sufficiently motivated to undergo the process of personalised risk assessment due to the number of samples required from the trio (including, blood and sperm samples) (table 2, CG13), whereas others who had recruited to PREGCARE did not report this as an issue, commenting that PREGCARE couples ‘*were quite happy to provide anything that would help to work things through’* (GCr20).

Essentially, practitioners said that if PREGCARE could refine the recurrence risk downwards, this would offer most couples the reassurance they seek. It would add clarity and evidence-based information to clinic consultations, provide justification for risk management, and release couples from unnecessary invasive testing. When the personalised risk was raised to 10% or above, practitioners reflected that although not offering reassurance through a reduction in risk, the PREGCARE outcome could justify access to further interventions on the NHS. However, in cases where PREGCARE testing identified maternal mosaicism—in which case the recurrence risk would be unquantifiable due to the inaccessibility of the oocytes—practitioners were concerned that this would be unhelpful and add to couples’ uncertainty. As CG08 observed: *“If it’s high risk but not quantifiable, then you’re back into the fudge figure”* (table 2, CG15). Practically, detection of maternal mixed mosaicism presents a challenging situation for counselling which should warrant extra-care in future pregnancies, even if the risk is not directly quantifiable, it is substantial. However, PGT-M is not suitable for these couples due to the presence of the DNM at low levels in maternal plasma.

Finally, when considering implementation, participants raised the importance of timing; when to inform patients about PREGCARE. DNM recurrence risk information is currently given in appointments focused on conveying a diagnosis for the affected child. Subsequent follow-up is often patient-led and triggered by a new pregnancy (table 2, CG14). Additionally, practitioners described how pregnancy brings time pressures for both practitioners and patients. As CG12 remarked: ‘*The difficulty is people come back pregnant and then there’s no time to offer it’* (table 2, CG19). Practitioners reflected that for PREGCARE to be useful, it would need to be discussed with couples fairly soon after diagnosis and to do this, clinic time would need to be allocated for pre-test and post-test genetic counselling (table 2, CG10, CG11). Needing ‘*careful handling and explanation*’ (CG02) and a level of ‘*genetic literacy*’ (GCr07), interviewees in our study thought that a precision risk assessment like PREGCARE would be better delivered within clinical genetics, rather than being a mainstream offering, in order to ensure that couples’ consent was better informed.

## Discussion

Gathering views of health professionals is critical for considering how and when new genomic tests are introduced into clinical practice. Clinical genetics professionals play a central role in informing and facilitating patient understanding and decision-making and therefore, their views and experience are key to the development of policy and guidelines. The above results suggest that precision risk assessment is appealing to clinical genetics practitioners. Participants generally regarded PREGCARE as beneficial in terms of giving them the confidence to clarify a couple’s specific circumstances, whether the risk was low and in refining the risk to improve risk management options for those with an increased risk.

The finding of a reassurance gap for DNM recurrence risk is consistent with the literature, which highlights the role of risk perception and risk acceptability when dealing with genetic risk information and uncertainty.^4 5 10–16^ Although effective reassurance is an essential medical task, not least because health anxiety impacts a patient’s understanding of information and can result in overuse of health services,^17–19^ in the context of genetic counselling low-risk results are not always reassuring.^13 20–22^ In this study, practitioners reported challenges in reassuring couples after a child is diagnosed with a genetic condition caused by a DNM and some couples remain incredibly anxious about making future reproductive decisions. They described trying to convey the risk as ‘low’ but that some couples perceived their risk as higher than other people because an event that is rare at the population level had already happened to them. This is consistent with other studies showing patient reactions driven by objective risk, but draw on life experience and heuristics, and are often ultimately binary in nature, translating a perceived risk to 50/50—either it will or will not happen again to them.^15 16 23^ Also, consistent with the study by Fumagalli *et al*,^7^ they told us that even when couples did perceive the risk to be small, their acceptability of any risk was often very low due to already having an affected child.^7^ Practitioners found it challenging that in the current setting they could not provide the reassurance these couples needed. They reflected that this uncertainty was difficult for some couples and, mirroring other studies,^24^ the expected burden of a second affected child could act as a driver for seeking PNT or opting for voluntary sterility.

There was also a reassurance gap for the practitioners themselves. They were aware that risk management and the advice they provide about reproductive decisions occur without information specific to a couple’s circumstance and wondered if they, as practitioners, had just been lucky not to have a recurrence among their cases so far. Concerns about accuracy and misleading information are consistent with other studies of practitioner views on delivering risk information, such as those on the early introduction of NIPT, in which interviewees stressed the importance of accuracy and the implications of inaccurate or uncertain results.^25–28^

The willingness to offer a next step—an invasive procedure such as chorionic villus sampling (CVS) or amniocentesis—to provide reassurance has also been observed in other studies. The study by Williams on fetal medicine practitioners’ perceptions of PNT reported they felt an imperative to ‘do something’ to help with the uncertainty driving patient anxiety.^29^ In the context of DNM recurrence, despite offering CVS/amniocentesis, the practitioners in our study did not regard it as medically necessary based on the objective (generic) risk. Mirroring other studies, they regarded NIPT/NIPD—relying on a blood sample from the pregnant woman for cell-free fetal DNA present during pregnancy—as better means of offering and obtaining reassurance^25 26 30–36^ and expressed frustration that these non-invasive options are not available to couples on the NHS based on the DNM generic recurrence risk. Of note, NIPD requires development of a custom assay for each DNM which can be challenging to design in a time-pressured situation of an ongoing pregnancy.

Taking place prior to a new pregnancy, practitioners thought that using PREGCARE during routine reproductive counselling following the diagnosis of a DNM would avoid leading couples unnecessarily into considerations of miscarriage risk and the distress of undergoing invasive procedures/termination of a wanted pregnancy for the purpose of reassurance.

The existing literature indicates that NIPD and other non-invasive options should be viewed critically.^19 37^ Likewise, our interviewees expressed concerns about anxiety driving some pregnant couples to take any test available, resulting in unnecessary and often expensive procedures.^13 25 34 38^ Some researchers have also taken issue with considerations of prenatal investigations such as ultrasound focusing only on the benefits^19^ while others have drawn attention to the decisional burden placed on patients with any prenatal risk management procedure because patients must consider the risks and benefits of the options offered, and they are drawn into considering new risk scenarios.^4^ Burton-Jeangros *et al* referred to this as ‘manufactured uncertainty’.^13^ Furthermore, although the offer of an unsolicited test may not be an impediment for making an autonomous choice, the moral significance of the offer is inseparably bound by the social context in which it is offered and it may be difficult for women or couples to decline such ‘options’ once they have been put forward by a health practitioner.^23 39^

The practitioners interviewed assumed the couples who did not return to clinic to discuss prenatal options were reassured by the generic recurrence risk provided. However, they also told us that there is no routine follow-up with these couples and their reproductive outcomes are unknown. Assuming reassurance for those who do not seek an ‘options’ discussion may be misleading. Perceived acceptability affects the interpretation of a given risk more than the objective probability of an event occurring—this also applies to risk management options such as non-invasive testing.^7^ For example, in an earlier US interview study of couples with children diagnosed with genetic conditions caused by DNMs (n=40), two-thirds of parents avoided choosing between troubling outcomes by not pursuing future childbearing (over two-thirds were still within childbearing age). This was due to low acceptability for the prenatal options at the time of the study (CVS or amniocentesis) and wanting to avoid the potential of being confronted with the choice of termination or continuation with an affected pregnancy.^6^ Interviewees in our study thought that PREGCARE, as a preconception tool, would avoid leading those couples stratified into categories with very low risk into unnecessary consideration of termination.

Further consistencies with the literature were found in the preparation of couples for undergoing assessment using a novel technique or technology. Here, the emphasis was on the importance of specialist handling. As in the research interviews with genetic counsellors regarding NIPT for sexing in relation to sex-linked conditions,^25^ the majority of participants in our study voiced a preference for PREGCARE to be delivered through clinical genetics. Similarly, a range of UK health practitioners in a qualitative study of NIPD^38^ also advocated for specialist providers, skilled in pre-test and post-test counselling. This interest in professional gatekeeping, also seen more broadly,^40^ represents a desire to uphold ethical standards and was accompanied by some anxiety about the capacity of other specialists to provide couples with sufficient pre-test counselling for informed consent. Mirroring studies on the introduction of NIPT/NIPD,^25 28 38 41^ our participants also appeared to have reservations about patient understandings of a novel test. Our data also suggest that discussing a range of possible test outcomes is an integral part of genetic counselling practice and offering PREGCARE in the context of reproductive decision-making would not pose a different sort of counselling challenge, although requiring additional explanatory training. The desire for clear guidelines and appropriate training is consistent with practitioners’ reactions to other new technologies.^25 38^

### Study limitations

Every effort was made to recruit both CGs and GCrs for this study. Although the former did outweigh the latter in the final sample (14:6), this likely reflects the starting point for discussing DNM recurrence risk with parents, which is a consultant-led diagnosis appointment for the family of the affected child. As a qualitative study, the results of this study are not generalisable and practitioners’ views on how couples would react to personalised information are hypothetical, although based on extensive clinical experience. Further qualitative research to elicit the perspective of couples themselves is planned.

### Study implications

This study has produced some tentative recommendations. PREGCARE was generally regarded as more beneficial than relying on generic risk, for both practitioner and couples. Sufficient clinic time would be needed for pre-test genetic counselling to ensure informed consent, support understanding and prepare couples for the range of possible outcomes. Result-giving would also require clinic time, especially when mosaicism is identified. Due to the skills required, the participants interviewed felt that PREGCARE should be accessed via clinical genetics in the first instance, or other services with embedded genetic counselling practitioners. Additionally, they regarded mosaicism as a complicated process for patients to understand and thought training and resources, such as the graphical representation of the seven scenarios presented in the PREGCARE study reports returned to referring consultants (see figure 1), would be beneficial. We note that the choice of 10% recurrence risk to qualify for NHS-funded PGT-M is biologically poorly defined and, in conjunction with our findings here and in the PREGCARE study, may need to be reconsidered in the light of our work.

## Data Availability

Data are available on reasonable request.
